# *Escherichia coli* Fails to Efficiently Maintain the Activity of an Important Flavin Monooxygenase in Recombinant Overexpression

**DOI:** 10.3389/fmicb.2017.02201

**Published:** 2017-11-13

**Authors:** Sofia Milker, Leticia C. P. Goncalves, Michael J. Fink, Florian Rudroff

**Affiliations:** ^1^Institute of Applied Synthetic Chemistry, TU Wien, Vienna, Austria; ^2^Department of Chemistry and Chemical Biology, Harvard University, Cambridge, MA, United States

**Keywords:** biotechnology, *Escherichia coli*, redox biocatalysis, Baeyer–Villiger monooxygenase, flavin, enzyme stability

## Abstract

This paper describes the measurement and analysis of *in vivo* activity and stability of cyclohexanone monooxygenase from *Acinetobacter* sp. NCIMB 9871 (CHMO), a model Baeyer–Villiger monooxygenase, in the recombinant host *Escherichia coli*. This enzyme was often described as poorly stable *in vitro*, and has recently been found to deactivate rapidly in the absence of its essential cofactors and antioxidants. Its stability *in vivo* was scarcely studied, so far. Under conditions common for the overexpression of CHMO we investigated the ability of the host to support these properties using metabolomics. Our results showed that *E. coli* failed to provide the intracellular levels of cofactors required to functionally stabilize the enzyme, although the biocatalyst was produced in high concentration, and was invariably detected after protein synthesis had stopped. We thus infer that biotechnological applications of CHMO with this host relied on a residual activity of approximately 5-10%. Other microorganisms might offer a more efficient solution for recombinant production of CHMO and related enzymes.

## Introduction

Flavin-dependent monooxygenases (FMOs), and especially Baeyer–Villiger monooxygenases (BVMOs), are useful biocatalysts for synthetic purposes because of their promiscuous acceptance of substrates (Torres Pazmiño et al., 2010; Bornscheuer et al., 2012; Fink et al., 2012; Bučko et al., 2016), and their mostly excellent stereoselectivity. Their dependence on oxygen as a terminal oxidant, and high activity at ambient temperature, can also offer technical advantages over (in)organic-synthetic methods (Bučko et al., 2016). A prominent member of this class of enzymes with a reportedly broad scope of substrates is cyclohexanone monooxygenase from *Acinetobacter* sp. NCIMB 9871 (CHMO) (Donoghue et al., 1976). Recently, it was found to be highly unstable in a cell-free setting (Van Beek et al., 2014). Such instability is likely common to many BVMOs, with few exceptions originating from thermophile organisms [PAMO (Fraaije et al., 2005) and TmCHMO (Romero et al., 2016)]. Genetic engineering has been used to enhance the stability and activity of CHMO *in vitro*: the introduction of stabilizing disulfide bonds led to an augmented thermodynamic stability by increasing the melting temperature of CHMO by 6°C (Van Beek et al., 2014), or 5°C (Schmidt et al., 2015). Still, even the improved variants showed poor kinetic stability (e.g., 33% residual activity after 24 h); the wild-type enzyme had a half-life of less than 2 min (Goncalves et al., 2017). As a consequence, even though CHMO and other BVMOs are chemically superior to other methods for the Baeyer–Villiger oxidation, their poor operational stability hinders applications on a scale relevant for the manufacture of fine or bulk chemicals (Balke et al., 2012). Only one industrial process (Bong et al., 2013) using a BVMO has been described, catalyzing the final step in the synthesis of esomeprazole (the API in the proton-pump inhibitor Nexium) on a multi-10 g scale by applying a heavily mutated CHMO variant (41 mutations).

A supposedly general solution for the lack of stability, or mitigation of its effects, was long seen in the use of recombinant whole-cell biocatalysts, e.g., *Escherichia coli*. Hypothetically, the maintenance of physiologically compatible conditions by the homeostatic cell would provide a stabilizing environment for fragile enzymes, such as CHMO. Failing that, the continuous re-synthesis of the enzyme, along with essential, spent cofactors, would still enable an efficient and conveniently usable system. For BVMOs, and specifically for CHMO, these assumptions are largely unchallenged, and were never quantitatively proven. Previous reports analyzed the effects of substrate and product inhibition, oxygen limitation, temperature, and pH on the operational stability of BVMOs as a whole-cell system (Doig et al., 2002, 2003; Baldwin and Woodley, 2006; Baldwin et al., 2008). It had been observed that a high intracellular concentration of NADPH was beneficial for the activity of CHMO (Walton and Stewart, 2004), but the mechanistic link between activity and operational stability of the BVMOs was hypothesized clearly, nor established experimentally.

In an *in vitro* study, we recently identified the following factors as influential in the deactivation of BVMOs *in vitro*: concentrations of (i) the BVMO itself, (ii) of the reduced cofactor NADPH, and (iii) of the coenzyme FAD (Goncalves et al., 2017). Supporting these results, we recently found by kinetic modeling that CHMO was the rate-limiting enzyme in a reaction cascade *in vivo* (Milker et al., 2017), even though it had the highest specific activity of the three sequential catalysts. We concluded that the low concentration of active CHMO was likely caused by an insufficient supply of NADPH and FAD in the cell. This study challenges the assumption that *E. coli* provides a beneficial environment for CHMO, or FMOs in general.

## Materials and Methods

### *E. coli* CHMO Batch Cultivation

*Escherichia coli* was cultivated in modified M9^∗^ minimal medium supplemented with 100 μg mL^-1^ ampicillin (For medium composition see Supplementary Information). Precultures were inoculated from late exponential phase-harvested, permanent cultures (-80°C, 1:100), and were grown for 16 h in a volume of 30 mL in non-baffled 500 mL Erlenmeyer flasks at 37°C and 350 rpm in an orbital shaker (InforsHT Multitron 2 Standard). The fermentation was performed at 37°C in a 2 L RALF fermenter (Bioengineering, Inc.) with a filling volume of 1 L. The stirring speed of the impeller turbines was set to 1000 rpm and the aeration rate kept constant at 1.5 vvm with air control by MX4/4 (DASGIP). The pH value was adjusted to 6.5 with 3 M NaOH and 3 M H_3_PO_4_, and inoculation was done at a ratio of 1:50 with the preculture. The off-gas analysis was performed with a GA4 off-gas analyzer (DASGIP). When foaming occurred antifoaming agent Antifoam O-30 (100 μL, Sigma–Aldrich) was added. At OD = 1, the bioreactor was cooled down to 20°C and the CHMO expression was induced with IPTG (100 μM, final concentration).

### Cell Density

The cell density of cultures was measured *via* absorbance at 590 nm in a spectrophotometric cell before the culture liquid was used for subsequent assays. The first sample was taken directly after inoculation. Therefore, 1 mL of the reaction culture was sampled every hour, and if necessary, diluted in a ratio of 1:10 with the medium to stay within the linear range (up to 0.7) of the apparatus (WPA colorwave CO7500 Colorimeter). We used the following relation: 1 OD = 0.43 g DCW^-1^ to determine the dry cell weight (Walton and Stewart, 2004).

### Cell Physiology

Samples for the analysis of the cell physiology were taken every hour. One mL of culture was transferred to a 1.5 mL Eppendorf reaction tube on ice and centrifuged (5000 × *g*, 10 min). The supernatant was filtered with a 0.2 μm syringe filter and stored at -20°C until HPLC analysis (Nexera Shimadzu). The supernatant was analyzed with a refractive index (RI) detector and photodiode array (PDA) detector for quantification of the analytes and an electrospray ionization (ESI) ion source with a quadrupole mass analyzer for additional confirmation of the substances (LC–MS 2020 Shimadzu). Separation was performed with an ROA-Organic Acid H+ (8%) column (300 mm × 7.8 mm, Phenomenex) with an isocratic flow of 0.4 mL min^-1^ 5 mM formic acid in water.

### Quantification of Soluble CHMO

The quantification of soluble CHMO was performed as single measurements per time point. The volume of the sample was adjusted, so that after centrifugation and the washing steps, the re-suspended cells (0.5 mL) would have an OD at 590 nm of 7.0 to later on have a quantifiable amount of CHMO. The sample was at first centrifuged (5000 × *g*, 10 min) and the resulting pellet was washed with 1 mL 50 mM Tris-HCl buffer, pH 7.5, then centrifuged again (5000 × *g*, 10 min), and stored at -20°C until further workup.

For cell lysis, the pellets were re-suspended in 0.5 mL 50 mM Tris-HCl, pH 7.5, containing 0.1 mM phenylmethylsulfonyl fluoride (PMSF). Cells were placed on ice and sonicated in nine cycles (5 s pulse, 55 s break, amplitude 50%, Bandelin KE76 sonotrode connected to a Bandelin Sonopuls HD 3200 wave generator). Precipitates were removed by centrifugation (15000 × *g*, 15 min) and the clear supernatants containing the soluble proteome, including CHMO, were analyzed by SDS-PAGE (Laemmli, 1970). Every SDS-PAGE gel was additionally loaded with at least three, differently concentrated samples of pure CHMO (range: 0.4–2.5 μg) in order to quantify the soluble CHMO concentration in every sample (for preparation of purified enzyme: see Supplementary Information). The gels were stained with SimplyBlue SafeStain (Thermo Fisher) using the microwave method according to the manufacturer’s protocol, scanned with the Molecular Imager Gel Doc XR System (BioRad), and quantified with the Image Lab Software (BioRad).

### *E. coli* Metabolomics

The sampling procedure was repeated every hour as a single point, and every 2 h in triplicates unless stated otherwise. Metabolomics samples were taken using a fast filtration sampling procedure, as described elsewhere (Link et al., 2012) (for details see Supplementary Information).

### LC–MS/MS Measurement

Dry extracts were re-suspended in water (100 μL) and centrifuged (12000 × *g*, 3 min). The supernatants were used for subsequent analyses. Separation was achieved with a Luna-NH_2_ HPLC column (150 mm × 2 mm, 3 μm particle size, 100 Å pore size, Phenomenex) using a binary gradient method (Solvent A: acetonitrile/Solvent B: 10 mM ammonium acetate, pH 9.9). Gradient parameters were as follows: 0–24 min: 20–100% B; 25–34 min: 100% B; 35 min: 20% B. Detection was performed with a tandem mass spectrometry detector with an ESI ion source (Shimadzu LCMS-8040) in multiple reaction monitoring (MRM) mode (Lorenz et al., 2011). Peak areas were normalized to fully ^13^C-labeled internal standards and absolute quantification of metabolites was achieved with linear calibration curves of the standards. Finally, concentrations were normalized to the amount of biomass. For MRM fragments see Supplementary Table S2.

### Activity Measurements *in Vivo*

Bicyclo[3.2.0]hept-2-en-6-one was purchased from Sigma–Aldrich and used as obtained. The activity measurements of CHMO in the batch culture were performed every 3 h. The first measurement was done 3 h post-induction with IPTG. For the activity measurement 5 mL of fermentation broth were transferred to a 250 mL non-baffled Erlenmeyer flask, and bicyclo[3.2.0]hept-2-en-6-one (8 mM, 1 M solution in dioxane) was added as substrate. The culture was placed on an orbital shaker at 20°C and 350 rpm to mimic the fermentation conditions in terms of aeration, mixing, and temperature. The first sample was taken immediately after substrate addition. Therefore, 100 μL of the culture broth were extracted with 500 μL ethyl acetate containing methyl benzoate (1 mM) as internal standard. The activity test was performed for 1 h; samples were taken every 15 min. The consumption of substrate as well as formation of the corresponding lactone was monitored by calibrated GC. The activity was normalized to the concentration of biomass, and to the soluble CHMO content to obtain the specific activity.

## Results

### Hypotheses and Experimental Design

We investigated the levels of and changes in concentrations of the following metabolites in a whole-cell overexpression system, which had been identified as crucial for CHMO’s activity and stability in earlier studies *in vitro* and *in silico*: CHMO itself, NADPH, FAD (Goncalves et al., 2017; Milker et al., 2017). We based our study on the following hypotheses:

(A) CHMO, produced by over-expression, is stable *in vivo* over the duration of a preparative experiment.
(B) CHMO is produced by *E. coli* to a sufficient concentration to achieve high specific activity of the whole-cell catalyst.
(C) The cofactors FAD and NADP(H) are produced by *E. coli* to sufficiently high concentrations to efficiently maintain a high specific activity of CHMO.
(D) *E. coli* is a suitable organism to act as an efficient whole-cell biocatalyst with FAD- and NADP(H)-dependent enzymes.

To test these hypotheses, we designed and conducted an experiment for the production of CHMO under controlled conditions in a bioreactor (pH, temperature, oxygen saturation) and performed the following analyses: we measured the intracellular concentration of CHMO (*via* SDS-PAGE and densitometry) and determined the activity of CHMO in a Baeyer–Villiger oxidation in a satellite culture to characterize the performance of the whole-cell biocatalyst. We determined the concentrations of the cofactors FAD and NADPH and their respective precursors (GXP, ATP), plus several indicative metabolites: amino acids (building blocks for the synthesis of CHMO) and compounds of the central carbon metabolism and tricarboxylic acid cycle (TCA) to monitor the physiology of *E. coli*. The sampling schedule is described in Supplementary Table S1.

### Activity, Stability, and Concentration of CHMO

**FIGURE 1:**
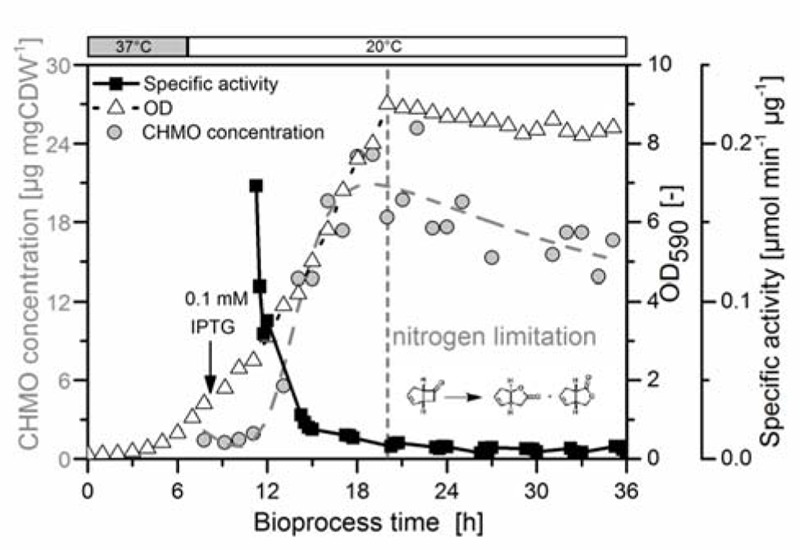
The specific activity of CHMO decayed rapidly to a basal level. *E. coli* BL21(DE3) pET22b::*chmo* was grown in minimal medium with sufficient aeration (see Supplementary Information, Supplementary Figure S2). The initial temperature of 37°C (exponential growth, growth rate at 37°C: 0.44 h^-1^) was lowered to 20°C after 8 h, shortly prior to induction with IPTG at a biomass concentration of 0.6 g dry cell weight per liter (g DCW L^-1^; OD_590_= 1.40). The culture reached nitrogen limitation after 20 h (maximum biomass concentration: 3.87 g DCW L^-1^). In the stationary phase the biomass decreased slightly to the final value of 3.61 g DCW L^-1^. The concentration of CHMO was determined by SDS-PAGE (Supplementary Figure S3) with a maximum value of 20.9 μg mg DCW^-1^ after 19 h, and was empirically interpolated using a composite function (exponential growth–exponential decay, see Supplementary Information). The activity of the cells in a Baeyer–Villiger oxidation of bicyclo[3.2.0]hept-2-en-6-one was measured in a satellite culture, which was incubated at the same temperature and comparable aeration/mixing conditions as in the bioreactor (Klöckner and Büchs, 2012). The specific activity was obtained by GC analysis, and normalized to the dry cell weight and to the protein concentration.

We induced the production of CHMO with IPTG in the exponential phase, and held the culture stationary with >60 mM glucose for 15 h after reaching a limitation by the nitrogen (Supplementary Figure S1). The maximum concentration of soluble CHMO was measured after 19 h (20.9 μg mg DCW^-1^, 11 h after induction). This value decreased toward the end of the experiment to 15.2 μg mg DCW^-1^ (**Figure 1**) with a small exponential decay constant of approximately 0.04 (assuming first-order deactivation of the enzyme in the stationary phase). We thus observed a loss of 27% of the soluble CHMO. This observation led us to reject hypothesis A: CHMO was not stable over a time span common for a preparative experiment.

We also observed a fast decrease in the specific activity with rising concentration of soluble CHMO: the highest activity was observed at the first measurement (3 h after induction). The specific activity dropped by approximately 90% within the first 5 h, and then remained constant for the next 21 h (**Figure 1**). Despite the inverse relationship of the concentration of CHMO and its specific activity, *E. coli* produced enough CHMO to theoretically reach high specific activity, thus supporting hypothesis B.

### Concentration of Cofactors NADPH and FAD

Based on our analytical results from metabolomics (quantification by ^13^C-standards in MS analysis), we estimated the intracellular concentrations of NADPH and FAD, using a published value for the volume of 3.2 ± 1.2 × 10^-15^ L per cell (Volkmer and Heinemann, 2011).

**FIGURE 2:**
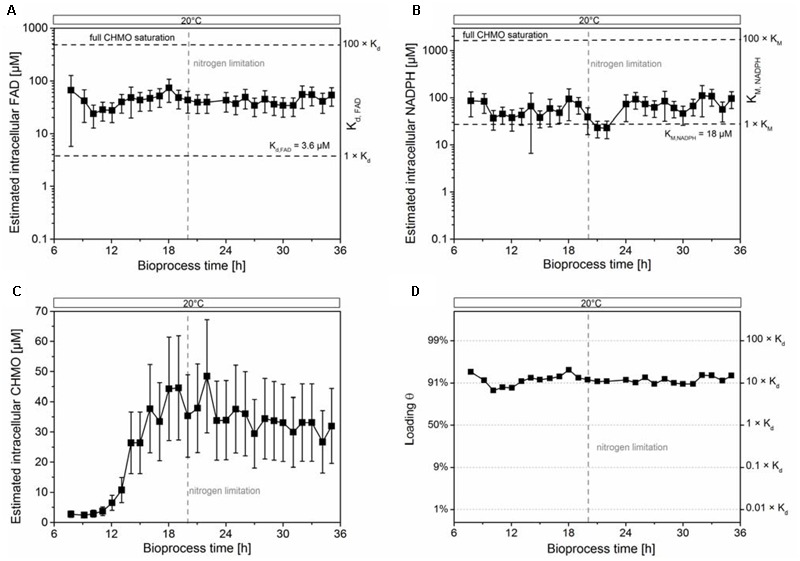
The intracellular concentrations of FAD and NADPH were insufficient to stabilize CHMO. The estimated intracellular concentrations of FAD and NADPH were stable **(A,B)**. The estimated intracellular concentration of CHMO **(C)** and the estimated saturation of CHMO with FAD **(D)** was stable under nitrogen limiting conditions within experimental error. The compounded errors were propagated from the uncertainty of the single components (see Supplementary Methods). Data are reported as mean ± propagated error (1 SD; see Supplementary Information).

We estimated the cytosolic concentration of CHMO, but the propagated error from the estimated volume did not allow the identification of a significant trend (see **Figure 2C**); the mean value was still used for calculations. We observed stable intracellular concentrations of FAD (40 ± 16 μM, **Figure 2A**) and NADPH (63 ± 29 μM, **Figure 2B**) after adaption to 20°C (increase in the uptake of oxygen, Supplementary Figure S2). Since the cells were extracted vigorously in sampling procedure, the measurement could not differentiate between the free and bound form of FAD (or of any other metabolite that would form non-covalent complexes), preventing the exact calculation of equilibrium concentrations. That notwithstanding, and given the uncertainty in the estimated values, we made the following, drastically simplifying assumptions to obtain an extreme boundary condition: (i) the total concentration of FAD in the cell is equal to the concentration of unbound FAD, and (ii) CHMO is the only FAD-dependent enzyme in the cell. This way, a hypothetical, but optimum scenario was created for the population of the CHMO⋅FAD complex (i.e., loading 𝜃), calculated using a plain logistic function:

$$\theta =\frac{\left[\text{CHMO}\cdot \text{FAD}\right]}{\left[{\text{CHMO}}_{\text{apo}}\right]}=1-\frac{1}{1+\frac{\left[\text{FAD}\right]}{{\text{K}}_{\text{d}}}}$$

with [CHMO⋅FAD] as the concentration of the CHMO⋅FAD complex, [CHMO_apo_] as the concentration of the CHMO apoenzyme, K_d_ as the dissociation constant of FAD (3.5 ± 0.6 μM; Goncalves et al., 2017), and [FAD] as the total intracellular concentration of FAD. Even under these theoretical conditions, the intracellular concentration of FAD was only sufficient to saturate on average 90–95% of CHMO with the coenzyme, far from thermodynamic saturation (>>99% at [FAD] > 100 × K_d_; **Figure 2D**). Given the large compounded uncertainty, and our extreme assumptions, we speculated that most of CHMO was actually present in its *apo* form. The decay of specific activity to a residual non-zero value corroborated the insufficient loading, indicating a rising lack of FAD with rising CHMO concentrations in the cell.

The estimated intracellular concentration of NADPH was sufficiently high to largely saturate the enzyme (0.73 × v_max_, K_M,NADPH_ = 18 μM; **Figure 2B**). Nevertheless, the concentration of NADPH was much lower than the level required for a strong stabilization, as earlier determined *in vitro.* There, a titer of >1 mM was found to have a large, beneficial effect on the thermodynamic and kinetic stability of CHMO (Goncalves et al., 2017). Together, these results could not clearly disprove hypothesis C on the basis of FAD; still, our data clearly showed that the cells could not provide enough of the coenzyme to saturate most CHMO molecules.

### Biosynthesis of the Cofactors

**FIGURE 3:**
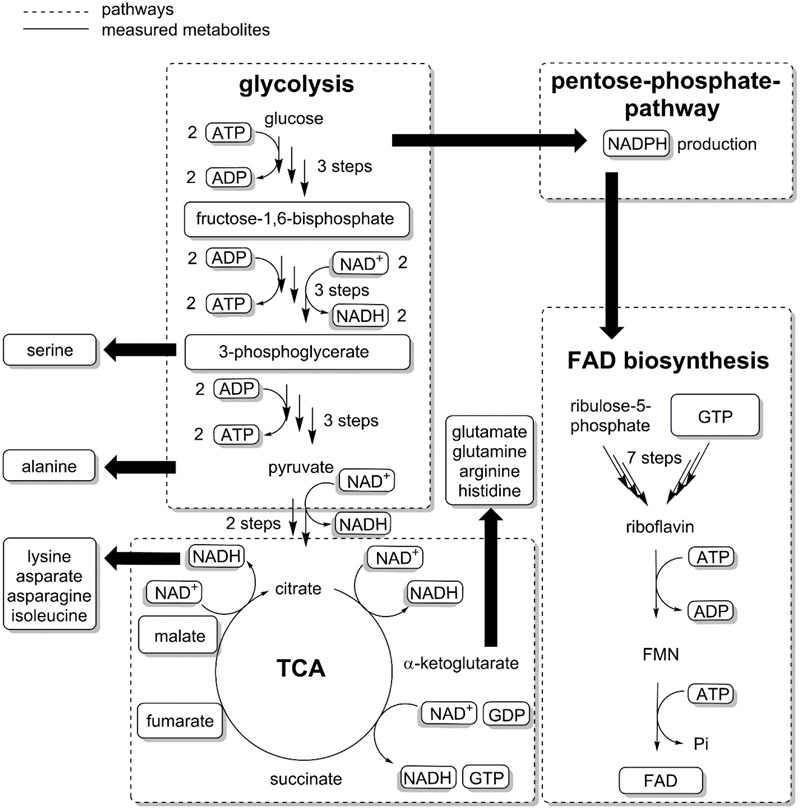
The biosynthesis of FAD requires GTP, ATP and ribulose-5-phosphate. GTP and Ribulose-5-phosphate are precursors for riboflavin, which after phosphorylation and reaction with ATP give FMN (flavin mononucleotide) and subsequently FAD. Metabolites were extracted from *E. coli* samples and measured with LC–MS/MS (see Supplementary Methods). Some metabolites were not quantified either because of low solubility (riboflavin) or insufficient resolution of the chromatographic method (ribulose-5-phosphate).

**FIGURE 4:**
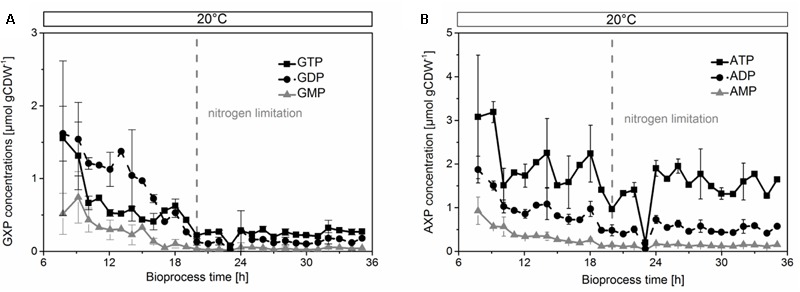
Concentrations of GXP **(A)** and AXP **(B)** decreased in the growing phase, but were stable during limitation by nitrogen. The concentrations were normalized by cell dry weight.

We then analyzed the metabolic network (**Figure 3**) to identify potential bottlenecks in the metabolic upkeep of active CHMO [specifically FAD, NADPH, and key metabolites that are required for their synthesis: guanosine and adenosine mono-, di-, and triphosphates (GMP, GDP, GTP, AMP, ADP, and ATP)]. We measured the concentrations of these biosynthetic precursors (**Figure 4**) to determine if a shortage thereof was limiting the availability of the cofactor to CHMO. GTP decreased already during the exponential growth phase of the cells (**Figure 4A**). After the culture had reached limitation by nitrogen, the concentration of the GXP pool was stable at approximately 0.300 ± 0.01 μmol gCDW^-1^; the three compounds of this pool can be interconverted by the host metabolism. The concentration of ATP was stable over the entire observed period (approximately 2.0 ± 0.2 μmol gCDW^-1^; **Figure 4B**); the concentrations of AMP and ADP decreased until the onset of the nitrogen limitation, and were then stable. We could not discern any apparent, meaningful correlation between the concentrations of ATP or GTP and FAD, or a bottleneck in their supply. The lack in FAD was thus not directly, or not obviously, caused by a poor supply of its precursors.

**FIGURE 5:**
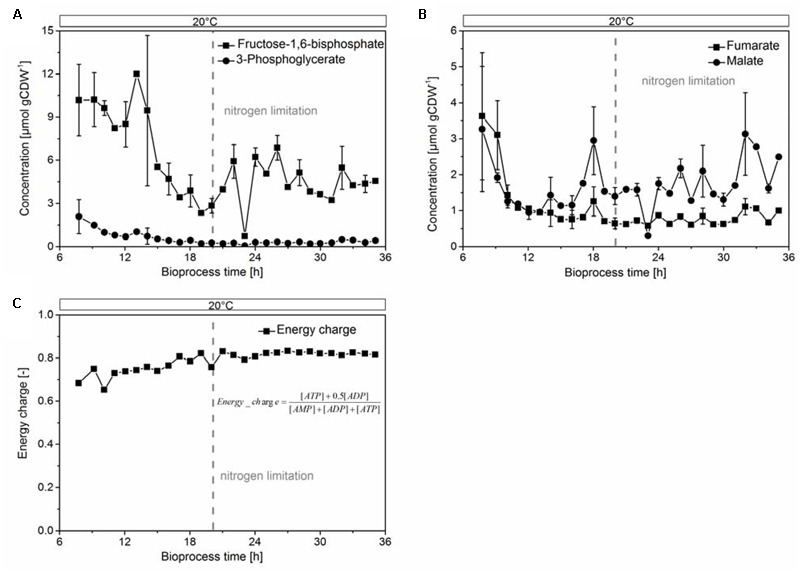
Glycolytic intermediates **(A)** fructose-1,6-bisphosphate, 3-phosphoglycerate; TCA intermediates **(B)** fumarate, malate, and the energy charge, calculated from the concentrations of ATP, ADP and AMP **(C)**, were stable under nitrogen-limited conditions.

To assess the proper function of the host organism, we investigated indicative parameters for growth, physiology, and biocatalytic performance of *E. coli*. We found an overall decrease of the glycolytic intermediates fructose-1,6-bisphosphate and 3-phosphoglycerate (**Figure 5A**), and the TCA components fumarate and malate (**Figure 5B**) after the onset of the nitrogen limitation. These metabolites remained constant within experimental error after physiological adaption of the culture to 20°C. The heavy metabolic burden of heterologous overexpression became apparent in an unfavorable value for the adenylate energy charge (**Figure 5C**) of 0.65 at the beginning of protein overproduction. It later stabilized to 0.83 under nitrogen limitation. This value reflected a balanced pool of adenosine phosphates (Chapman et al., 1971) and cell viability until the end of the cultivation. The protein production stopped when the culture reached the nitrogen limitation, which was reflected in the concentration curves of most amino acids. Specifically, the low concentrations of glutamine, glutamate, and aspartate after the nitrogen limitation prevented the organism from further synthesizing CHMO (or other proteins, Supplementary Figures S4D–F).

In summary, the host organism *E. coli* was found capable of maintaining the NADPH requirements of CHMO, providing a stable environment with sufficiently high levels of precursors for the protein itself, as well as for essential cofactors. However, due to the low concentration of FAD, it failed to fully saturate CHMO in overexpression. Although the experimental evidence cannot lead us to directly reject hypothesis D — all necessary compounds were invariably present to some extent—, it does not support the assumption that *E. coli* is a suitable host for the *efficient* maintenance of CHMO.

## Discussion

Our findings suggest that the paradigm of placing a poorly stable biocatalyst, such as CHMO, into a living cell to provide for a stabilizing environment lacks specific experimental evidence. In regard of demanding requirements to stabilize CHMO (high concentrations of the cofactors NADPH and FAD), we compared *in vitro* (Goncalves et al., 2017) and *in vivo* reaction modes, and provided substantial evidence that generic *in vivo* stabilization utterly failed with CHMO; we additionally infer that this host organism would also fail with similar enzymes. Under our controlled reaction conditions, the peak concentration of soluble CHMO decreased slightly, but without any meaningful correlation to the significant loss in activity (**Figure 1**). This finding is in contrast to a previous study, which identified the (unspecified) decay of CHMO itself as main reason for the loss of activity (Walton and Stewart, 2004). We found that the supply of NADPH in this standard *E. coli* strain was sufficiently high to allow the enzyme to operate efficiently (approximately 3 × K_M_), but it was still orders of magnitude below a stabilizing concentration (approximately 1 mM). Similarly, the intracellular concentration of FAD was insufficient to completely saturate the enzyme. We speculate that the processes of deactivation and synthesis of the target enzyme were competing, as long as the culture was growing, as a plausible reason for the drop in specific activity during that period. When synthesis ceased, in the final phase of the experiment, a constant residual activity of CHMO in the range of 5–10% was observed, likely resulting from a small fraction of holo-CHMO.

We followed the biosynthetic pathway for FAD, starting from GTP and ribulose-5-phosphate, an intermediate of the pentose phosphate pathway (PPP) (Abbas and Sibirny, 2011) (**Figure 3**). Nitrogen limitation caused a decrease in PPP metabolites (Brauer et al., 2006), and thus interfered with the supply of building blocks for FAD. These results were corroborated by our previous study on an enzymatic cascade, where the presence of a second, overexpressed flavin-dependent enzyme in *E. coli* was found likely to decrease the availability of FAD (Milker et al., 2017). Even under the extreme assumption that CHMO was the only FAD-dependent enzyme in *E. coli*, the host failed to provide enough of the cofactor to reach maximum efficiency. Moreover, the genome of *E. coli* encodes for approximately 80 flavin-dependent enzymes (Macheroux et al., 2011). They presumably all are essential for growth, or homeostasis, to unknown relative proportions, and are thus constantly competing for the pool of available, free FAD. Given the low stability of the CHMO⋅FAD complex, even an FAD-overproducing organism might not provide sufficient amounts for complete functional stabilization.

These results strongly suggest that the investigated organism *E. coli* BL21 (DE3) is incapable of efficiently maintaining the activity of CHMO, an important FMO, under commonly used conditions for overexpression. Overall, its performance as a stabilizing host would only be sufficient for NADPH-dependent enzymes, but not if they also rely on FAD, with an affinity to that of CHMO. Analysis of the central carbon metabolism and the biosynthetic pathway for FAD indicated that the scarcity was a deep-rooted and widely distributed problem created by the metabolic network. Other well-explored species for heterologous expression, but with reportedly better flavin production (e.g., *Pichia pastoris, Bacillus subtilis, Candida famata*) (Abbas and Sibirny, 2011) might thus offer a more suitable artificial habitat for CHMO.

## Author Contributions

MF and FR conceived the research and planned the experiments. All authors performed the culturing experiment and analyzed the data. SM performed the instrumental analyses. LG performed the protein purification. All authors co-wrote the manuscript and approved of the final version.

## Conflict of Interest Statement

The authors declare that the research was conducted in the absence of any commercial or financial relationships that could be construed as a potential conflict of interest.
