# Supplementary material for: Escherichia coli Fails to Efficiently Maintain the Activity of an Important Flavin Monooxygenase in Recombinant Overexpression
Source: Front Microbiol. 2017 Nov 13;8:2201. doi: 10.3389/fmicb.2017.02201 (PMC5693912; doi:10.3389/fmicb.2017.02201)
Supplement: Supplementary file 1 [file Data_Sheet_1.DOCX]

Supplementary Material

*Escherichia coli* fails to efficiently maintain an important flavin monooxygenase in recombinant over-expression

Sofia Milker^1^, Leticia C. P. Goncalves^1^, Michael J. Fink^1,2,^*, and Florian Rudroff^1,^*

^1^ Institute of Applied Synthetic Chemistry, TU Wien, Vienna, Austria

^2^ Department of Chemistry and Chemical Biology, Harvard University, Cambridge, MA, USA

*** Correspondence:** M.J.F. (mfink@gmwgroup.harvard.edu) and F.R. (florian.rudroff@tuwien.ac.at)

[1 Supplementary Methods 3](#_Toc492642931)

[1.1 Growth of bacterial cells for enzyme expression and isolation 3](#_Toc492642932)

[1.2 Enzyme purification 3](#_Toc492642933)

[1.3 Bacterial strain and culture conditions for recombinant CHMO synthesis 3](#_Toc492642934)

[1.4 *E. coli* metabolomics 4](#_Toc492642935)

[1.5 Fitting of experimental data 4](#_Toc492642936)

[1.6 Propagation of uncertainty for intracellular FAD, NADPH and CHMO concentrations, and the FAD loading 4](#_Toc492642937)

[1.8 Supplementary Data 6](#_Toc492642938)

[1.8.1 Supplementary Table S1. Time points for protein-, metabolomics- and *in vivo* CHMO activity samples. Biomass concentration was measured at every time point. 6](#_Toc492642939)

[1.8.2 Supplementary Table S2. Multiple reaction monitoring (MRM) precursor ions and fragments for metabolomics measurement. 7](#_Toc492642940)

[1.8.3 Supplementary Figure S1. Physiology analysis: glucose and pyruvate concentrations. 8](#_Toc492642941)

[1.8.4 Supplementary Figure S2. Off-gas analysis during the *in vivo* CHMO activity experiment. 9](#_Toc492642942)

[1.8.5 Supplementary Figure S3. SDS-PAGE gel for quantification of soluble CHMO in the cultivation of E. coli BL21(DE3)/pET22b::chmo 10](#_Toc492642943)

[1.8.6 Supplementary Figure S4. Intracellular concentration of selected amino acids. 11](#_Toc492642944)

# Supplementary Methods

## Growth of bacterial cells for enzyme expression and isolation

CHMO (cyclohexanone monooxygenase from *Acinetobacter calcoaceticus* NCIMB 9871) was expressed in *E. coli* strain BL21(DE3). Lysogeny broth (LB) medium (10 mL) supplemented with ampicillin (100 µg mL^-1^) was inoculated with single colonies containing the DNA coding regions for CHMO. These were grown over-night at 37 °C in an orbital shaker operated at 200 rpm. The cultures were transferred to a 2 L Erlenmeyer flask containing 500 mL of a LB/ampicillin medium, which was shaken at 200 rpm and 37 °C for approximately 2.5 h to a final optical cell density at 590 nm of 0.6–0.8. Isopropyl-β-D-thiogalactopyranoside (IPTG) was added to a concentration of 50 μM and flasks were incubated for 18–22 h at 20 °C. Cells were harvested by centrifugation (4000 × g, 15 min).

## Enzyme purification

Cell pellets were re-suspended in 50 mM Tris-HCl buffer, pH 8.0, containing phenylmethylsulfonyl fluoride (PMSF, 0.1 mM) and FAD (0.1 mM). Cells were placed on ice and sonicated using a Bandelin KE76 sonotrode connected to a Bandelin Sonoplus HD 3200 in 9 cycles (5 s pulse, 55 s break, amplitude 50%). Precipitates were removed by centrifugation (45 min, 15000 × g) and the clear supernatant containing the polyhistidine-tagged CHMO was loaded on a Ni^2+^-sepharose HP affinity column (5 mL, GE Healthcare bioscience) equilibrated with 50 mM Tris-HCl buffer, pH 8.0, containing 0.5 M NaCl. The column was washed with 7 column volumes 50 mM Tris-HCl buffer, pH 8.0, containing 0.5 M NaCl and 25 mM imidazol The CHMO was eluted in 4 column volumes with 250 mM imidazole in 50 mM Tris-HCl buffer, pH 8.0, containing 0.5 M NaCl. The CHMO containing fractions were identified by SDS-PAGE, pooled, desalted, washed with 50 mM Tris-HCl, pH 8.5, and concentrated by ultrafiltration by using ultracentrifugal tubes with a cut-off of 10 kDa. Protein concentrations were determined by the dye-binding method of Bradford using a pre-fabricated assay (BioRad) and bovine serum albumin as the calibration standard.

## Bacterial strain and culture conditions for recombinant CHMO synthesis

*E. coli* BL21 (DE3) was used as expression host for CHMO synthesis (pET22b–CHMO). For *E. coli* cultivation a modified M9* minimal medium (3 × (NH_4_)_2_ SO_4_ & 3 × MgSO_4_) was used.

The M9* medium contained the following substances: 2.4 g L^-1^ (NH_4_)_2_ SO_4_, 0.5 g L^-1^ NaCl, 7.5 g L^-1^ Na_2_HPO_4_ ⋅ 2H_2_O and 3.0 g L^-1^ KH_2_PO_4_. The following components were sterilized separately by filtration and then added to the main solution: 3 mL L^-1^ of 1 M MgSO_4_, 1 mL L^-1^ of 0.1 M CaCl_2_, 0.6 mL L^-1^ 0.1 M FeCl_3_ ⋅ 6H_2_O, 2 mL L^-1^ 1 mM thiamine ⋅ HCl and 10 mL L^-1^ of the trace element solution.

The trace element solution was sterilized by filtration and contained 0.18 g L^-1^ ZnSO_4_ ⋅ 7H_2_O, 0.12 g L^-1^ CuCl_2_ ⋅ 2H_2_O, 0.12 g L^-1^ MnSO_4_ ⋅ H_2_O, 0.18 g L^-1^ CoCl_2_ ⋅ 6H_2_O, 0.03 g L^-1^ H_3_BO_3_, 0.025 g L^-1^ Na_2_MoO_4_ ⋅ 2 H_2_O and 0.084 g L^-1^ Na_2_EDTA ⋅ 2 H_2_O.

Filter-sterilized glucose was added to a ﬁnal concentration of 20 g L^-1^.

All materials and media compounds were sterilized by autoclaving at 121°C for 20 min except for heat-sensitive aqueous solutions. These were sterilized by filtration either through 0.20 µm sterile syringe filters (VWR) for small volumes or sterile 0.20 µm bottle-top vacuum filters for big volumes of up to 1 L (Nalgene, Thermo Fisher Scientific).

## *E. coli* metabolomics

The sampling procedure was repeated every hour as a single point and every two hours in triplicates unless otherwise stated. Metabolomics samples were taken according to a literature protocol by a fast filtration sampling procedure (3). Therefore, the nitrocellulose filter was placed onto the filtration apparatus and moistened with M9 medium (2 mL) with or without nitrogen depending on if the metabolic sample was taken prior or after the culture has reached non-growing nitrogen limited conditions. Immediately after wetting the membrane filter (0.45 µm nitrocellulose MF Membrane Filters, Millipore), 2 mL sample solution was dispersed onto the filter and washed with 4 mL of the corresponding M9 medium (with or without nitrogen). It was important that the filter did not run dry. The filter was removed with tweezers, placed into a 15 mL centrifugation tube and frozen in liquid nitrogen. The samples were stored at -80°C until extraction. Two mL of the extraction solution (60:40 v/v ethanol/water) were preheated to 78°C in a water bath. U-^13^C internal standard(4) was added to the centrifugation tube with the filtered sample and the 2 mL extraction solution were added. The centrifugation tube was transferred to the water bath and incubated for 3 min at 78°C while vortexing every minute. The samples were stored at -40°C in precooled ethanol until centrifugation (12000 × g, 10 min, -9°C). The samples were dried at 30°C and 0.12 mbar to complete dryness in a vacuum concentrator and subsequently stored at -80°C until LC-MS/MS measurement.

## Fitting of experimental data

The data points of the concentration of CHMO, normalized by cell density, was empirically interpolated using a composite function (exponential growth–exponential decay) by non-linear fitting (OriginPro 2015) to obtain a first-order decay constant :$y=A_{2}+\frac{(A_{1}-A_{2})}{1+{(\frac{x}{x_{0}})}^{p}}+y_{0}+A_{3}e^{-xk}$, with A_1_ = ‑5.30, A_2_ = 6.60, A_3_ = 13.98, p = 10.84, k = 0.04 and y_0_ = ‑3.43.

## Propagation of uncertainty for intracellular FAD, NADPH and CHMO concentrations, and the FAD loading

Standard deviation for intracellular FAD:

$$\sigma_{FAD}= \sqrt{\left( \frac{\sigma_{FAD}}{[FAD]} \right)^{2}+\left( \frac{\sigma_{V_{cell}}}{V_{cell}} \right)^{2}{+ \left( \frac{\sigma_{{Cells}_{mL}}}{{Cells}_{mL}} \right)}^{2}}$$

Standard deviation for intracellular NADPH:

$$\sigma_{NADPH}= \sqrt{\left( \frac{\sigma_{NADPH}}{[NADPH]} \right)^{2}+\left( \frac{\sigma_{V_{cell}}}{V_{cell}} \right)^{2}{+ \left( \frac{\sigma_{{Cells}_{mL}}}{{Cells}_{mL}} \right)}^{2}}$$

Standard deviation for intracellular CHMO:

$$\sigma_{CHMO}= \sqrt{\left( \frac{\sigma_{CHMO}}{[CHMO]} \right)^{2}+\left( \frac{\sigma_{V_{cell}}}{V_{cell}} \right)^{2}{+ \left( \frac{\sigma_{{Cells}_{mL}}}{{Cells}_{mL}} \right)}^{2}}$$

Due to the analytical failure of common, simple methods for the propagation of uncertainty for the logistic function, the standard deviation for intracellular FAD loading of CHMO was estimated by calculating the extreme combinations of errors.

##

## Supplementary Data

### Supplementary Table S1. Time points for protein-, metabolomics- and *in vivo* CHMO activity samples. Biomass concentration was measured at every time point.

| **Incubation time [h]** | **Event** | **Protein sample** | **Metabolomic sample** | | **Activity sample** |
| --- | --- | --- | --- | --- | --- |
| 0.00 | Inoculation |  |  |  | |
| 0.02 |  |  |  |  | |
| 1.00 |  |  |  |  | |
| 2.00 |  |  |  |  | |
| 3.00 |  |  |  |  | |
| 4.00 |  |  |  |  | |
| 5.00 |  |  |  |  | |
| 6.00 |  |  |  |  | |
| 7.00 | Cooling to 20 °C |  |  |  | |
| 7.77 |  | X | Triplicate |  | |
| 8.12 | Induction, 0.1 mM IPTG |  |  |  | |
| 9.17 |  | X | Duplicate |  | |
| 10.12 |  | X | Triplicate |  | |
| 11.08 |  | X | Single point | X | |
| 12.07 |  | X | Triplicate |  | |
| 13.08 |  | X | Single point |  | |
| 14.07 |  | X | Triplicate | X | |
| 15.00 |  | X | Single point |  | |
| 16.05 |  | X | Triplicate |  | |
| 17.05 |  | X | Single point | X | |
| 18.03 |  | X | Triplicate |  | |
| 19.03 |  | X | Single point |  | |
| 20.00 |  | X | Triplicate | X | |
| 21.03 |  | X | Single point |  | |
| 21.13 | Glucose addition (20 mL, 50%) |  |  |  | |
| 22.02 |  | X | Triplicate |  | |
| 23.03 |  | X | Single point | X | |
| 24.02 |  | X | Triplicate |  | |
| 25.03 |  | X | Single point |  | |
| 26.03 |  | X | Triplicate | X | |
| 27.00 |  | X |  |  | |
| 27.12 |  |  | Single point |  | |
| 28.08 |  | X | Triplicate |  | |
| 29.05 |  | X | Single point | X | |
| 29.98 |  | X | Triplicate |  | |
| 31.05 |  | X | Single point |  | |
| 32.00 |  | X | Triplicate | X | |
| 33.00 |  | X | Single point |  | |
| 34.13 |  | X | Duplicate |  | |
| 35.08 |  | X | Single point | X | |

### Supplementary Table S2. Multiple reaction monitoring (MRM) precursor ions and fragments for metabolomics measurement.

| **Compound** | **Precursor (m/z)** | **1. Fragment (m/z)** | **2. Fragment (m/z)** | **3. Fragment (m/z)** | **Mode** |
| --- | --- | --- | --- | --- | --- |
| Cofactors | | | | | |
| ^13^C FAD | 813.05 | 358.00 | 141.15 | 456.05 | positive |
| ^12^C FAD | 786.05 | 348.00 | 439.05 | 136.15 | positive |
| ^13^C NAD | 684.70 | 141.00 | 438.05 | – | positive |
| ^12^C NAD | 663.70 | 136.00 | 428.05 | – | positive |
| ^13^C NADH | 687.20 | 670.10 | 530.50 | – | positive |
| ^12^C NADH | 666.20 | 649.10 | 514.05 | – | positive |
| ^13^C NADPH | 767.00 | 750.00 | 141.00 | 313.05 | positive |
| ^12^C NADPH | 746.00 | 729.00 | 136.00 | 302.05 | positive |
| AXP and GXP | | | | | |
| ^13^C AMP | 357.90 | 141.05 | 97.05 | – | positive |
| ^12^C AMP | 347.90 | 136.05 | 97.05 | – | positive |
| ^13^C ADP | 437.70 | 141.05 | 358.05 | – | positive |
| ^12^C ADP | 427.70 | 136.05 | 348.05 | – | positive |
| ^13^C ATP | 518.00 | 145.95 | 438.00 | 97.05 | positive |
| ^12^C ATP | 508.00 | 135.95 | 428.00 | 97.05 | positive |
| ^13^C GMP | 373.90 | 157.00 | 97.05 | – | positive |
| ^12^C GMP | 363.90 | 152.00 | 97.05 | – | positive |
| ^13^C GDP | 453.90 | 157.00 | 97.00 | – | positive |
| ^12^C GDP | 443.90 | 152.00 | 97.00 | – | positive |
| ^13^C GTP | 534.00 | 157.05 | 97.05 | – | positive |
| ^12^C GTP | 524.00 | 152.05 | 97.05 | – | positive |
| Glycolytic and TCA intermediates | | | | | |
| ^13^C F-1.6-BP | 344.90 | 96.85 | 79.10 | – | negative |
| ^12^C F-1.6-BP | 338.90 | 96.85 | 79.10 | – | negative |
| ^13^C Fumarate | 119.00 | 74.10 | 28.15 | – | negative |
| ^12^C Fumarate | 115.00 | 71.10 | 27.15 | – | negative |
| ^13^C Malate | 137.00 | 119.00 | 92.10 | – | negative |
| ^12^C Malate | 133.00 | 115.00 | 89.10 | – | negative |
| ^13^C 3-PG | 188.00 | 97.00 | 79.10 | – | negative |
| ^12^C 3-PG | 185.00 | 97.10 | 79.10 | – | negative |
| Amino acids | | | | | |
| ^13^C Alanine | 92.90 | 45.20 | 46.00 | 29.00 | positive |
| ^12^C Alanine | 89.90 | 44.20 | 45.00 | 28.00 | positive |
| ^13^C Arginine | 180.70 | 61.10 | 121.10 | – | positive |
| ^12^C Arginine | 174.70 | 60.10 | 116.10 | – | positive |
| ^13^C Asparagine | 136.80 | 76.05 | 90.00 | – | positive |
| ^12^C Asparagine | 132.80 | 74.05 | 87.00 | – | positive |
| ^13^C Aspartate | 136.00 | 91.05 | 119.05 | – | negative |
| ^12^C Aspartate | 132.00 | 88.05 | 115.05 | – | negative |
| ^13^C Glutamate | 153.20 | 88.05 | 60.05 | – | positive |
| ^12^C Glutamate | 148.20 | 84.05 | 56.05 | – | positive |
| ^13^C Glutamine | 151.70 | 88.05 | 135.05 | – | positive |
| ^12^C Glutamine | 146.70 | 84.05 | 130.05 | – | positive |
| ^13^C Histidine | 161.60 | 115.05 | 87.05 | – | positive |
| ^12^C Histidine | 155.60 | 110.05 | 83.05 | – | positive |
| ^13^C Isoleucine | 137.90 | 91.10 | 45.10 | – | positive |
| ^12^C Isoleucine | 131.90 | 86.10 | 44.10 | – | positive |
| ^13^C Lysine | 152.90 | 89.10 | 136.10 | – | positive |
| ^12^C Lysine | 146.90 | 84.10 | 130.10 | – | positive |
| ^13^C Serine | 109.20 | 91.10 | 73.15 | – | positive |
| ^12^C Serine | 106.20 | 88.10 | 70.15 | – | positive |

AMP/ADP/ATP: Adenosine mono/di/triphosphate; GMP/GDP/GTP: Guanosine mono/di/triphosphate; 3-PG: 3-phosphoglycerate, F-1,6-BP: Fructose-1,6-bisphosphate

### **Supplementary Figure S1. Physiology analysis: glucose and pyruvate concentrations.**


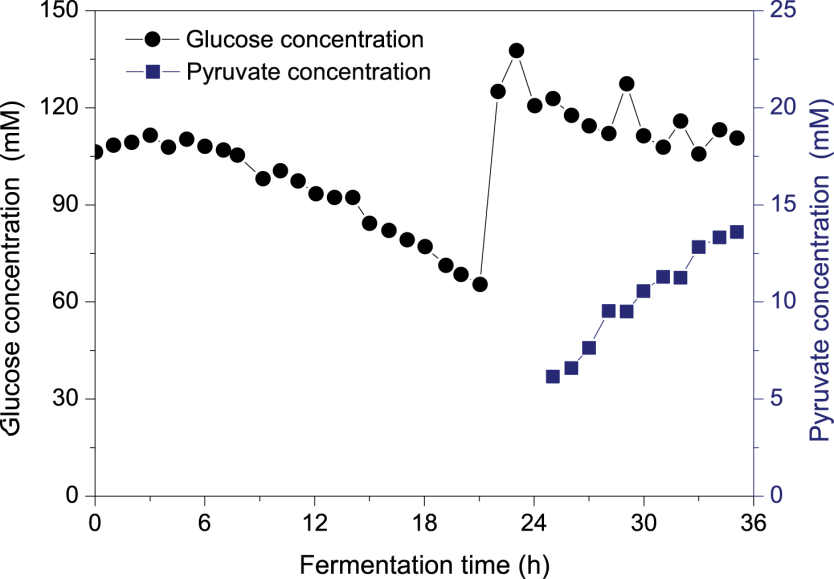


***E. coli* BL21(DE3)/pET22b::*chmo* physiology analysis: glucose and pyruvate concentrations.** Glucose and pyruvate concentrations in the supernatant of the culture plotted over time. After 20 h, glucose was pulsed to the culture.

### Supplementary Figure S2. Off-gas analysis during the *in vivo* CHMO activity experiment.


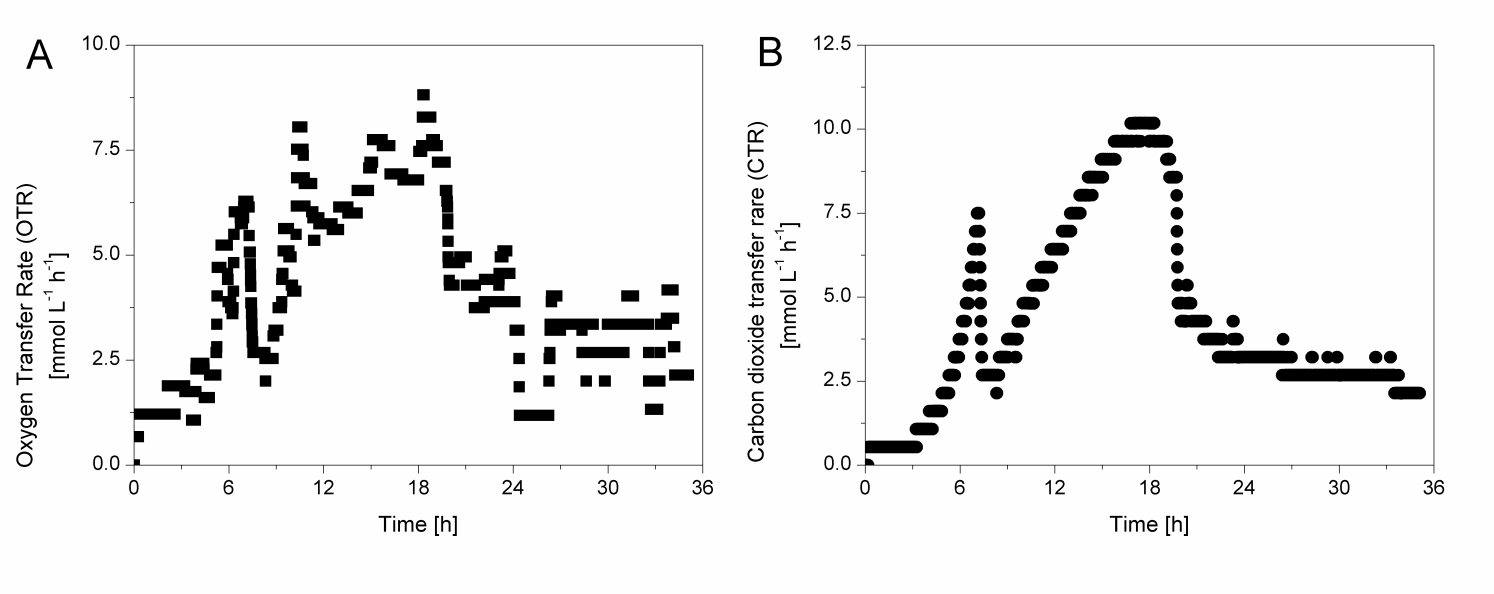


**Off-gas analysis.** (A) oxygen transfer rate (OTR) and (B) carbon dioxide transfer rate (CTR) plotted over cultivation time. The 2 L bioreactor was operated at 1 L filling volume with 1000 rpm stirring frequency and 1.5 vvm specific aeration rate. The temperature was 37°C before induction. Shortly before induction with 0.1 mM IPTG after 8 h, the bioreactor was cooled down to 20°C.

### Supplementary Figure S3. SDS-PAGE gel for quantification of soluble CHMO in the cultivation of E. coli BL21(DE3)/pET22b::chmo


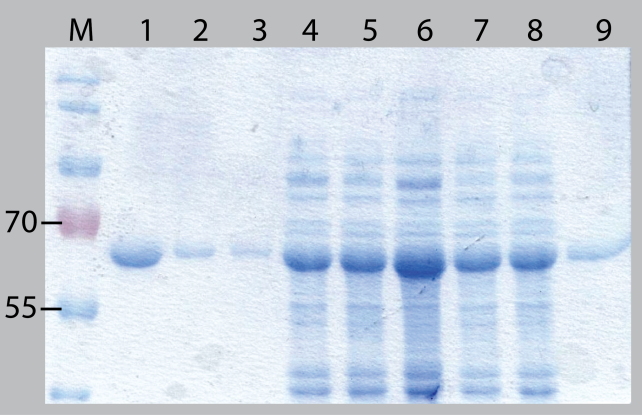


**SDS-PAGE gel for quantification of soluble CHMO in the cultivation of *E. coli* BL21(DE3)/pET22b::*chmo*.** (M) marker: PageRuler™ Prestained Protein Ladder; (1) 2.5 µg purified CHMO; (2) 0.60 µg purified CHMO; (3) 0.40 µg purified CHMO; (4) – (8) culture samples; (9) 1.2 µg purified CHMO.

### Supplementary Figure S4. Intracellular concentration of selected amino acids.


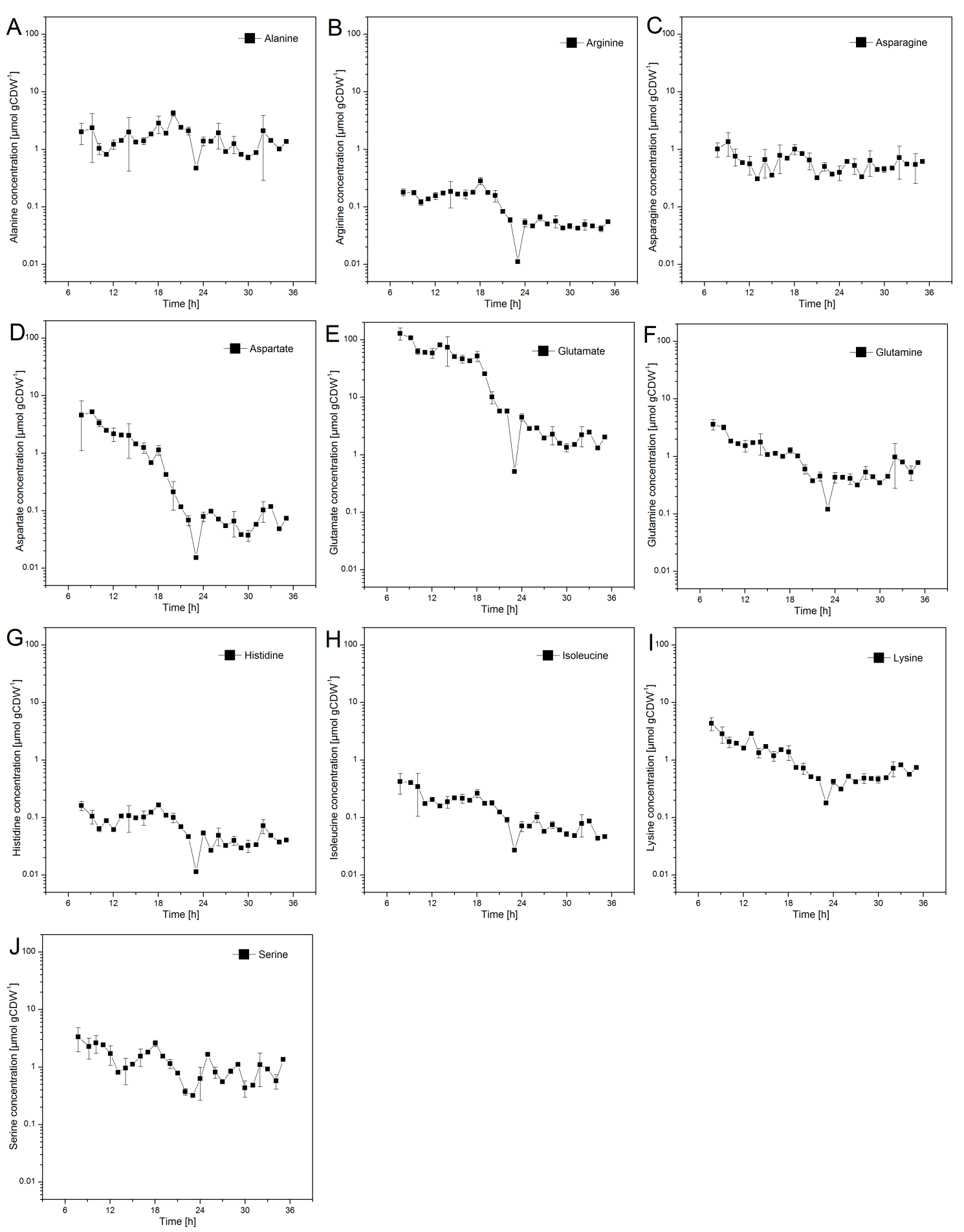


**Intracellular concentration of several amino acids in the cultivation of *E. coli* BL21(DE3)/pET22b::*chmo*.** The concentration of the metabolites (A) alanine, (B) arginine, (C) asparagine, (D) aspartate, (E) glutamate, (F) glutamine, (G) histidine, (H) isoleucine, (I) lysine, (J) serine is normed by gram cell dry weight (gCDW) for each time point. The measurement was performed every hour as single sample and every second hour in triplicates.
